# Unsupervised Trademark Retrieval Method Based on Attention Mechanism

**DOI:** 10.3390/s21051894

**Published:** 2021-03-08

**Authors:** Jiangzhong Cao, Yunfei Huang, Qingyun Dai, Wing-Kuen Ling

**Affiliations:** 1 School of Information Engineering, Guangdong University of Technology, Guangzhou 510006, China; hyfyunfei0325@gmail.com (Y.H.); yongquanling@gdut.edu.cn (W.-K.L.); 2 Guangdong Provincial Key Laboratory of Intellectual Property and Big Data, Guangdong Polytechnic Normal University, Guangzhou 510006, China; dqy@gpnu.edu.cn

**Keywords:** trademark retrieval, instance discrimination, attention mechanism, local cross-channel interaction

## Abstract

Aiming at the high cost of data labeling and ignoring the internal relevance of features in existing trademark retrieval methods, this paper proposes an unsupervised trademark retrieval method based on attention mechanism. In the proposed method, the instance discrimination framework is adopted and a lightweight attention mechanism is introduced to allocate a more reasonable learning weight to key features. With an unsupervised way, this proposed method can obtain good feature representation of trademarks and improve the performance of trademark retrieval. Extensive comparative experiments on the METU trademark dataset are conducted. The experimental results show that the proposed method is significantly better than traditional trademark retrieval methods and most existing supervised learning methods. The proposed method obtained a smaller value of NAR (Normalized Average Rank) at 0.051, which verifies the effectiveness of the proposed method in trademark retrieval.

## 1. Introduction

As an important intellectual property, trademarks play an important role in social and economic development. In many countries, trademark owners register trademarks with intellectual property agencies to legalize them and protect their rights. Currently, there are nearly 8.5 million trademark applications worldwide, and the number of trademark applications is increasing at a rate of 13.6% per year [1]. To judge whether the trademark is infringed or not, the relevant experts evaluate the similarity of the trademark. The effective and efficient retrieval of trademarks has become the bottleneck to the management, protection, and application of trademarks. In the past, trademark retrieval was carried out in the form of a “classification number”, which divides trademarks into different kinds manually, however, such a method is time-consuming and has low efficiency since the important information carrier for trademarks are images. In order to solve the problem of retrieval work, researchers began to use content-based image retrieval methods to avoid deviations caused by text descriptions, thereby capturing more accurate trademark feature information.

The key of trademark retrieval is the extraction and measurement of trademark image features. The accuracy of trademark feature extraction directly affects the subsequent retrieval results. In traditional trademark retrieval methods, people are more inclined to extract features through the shallow visual features of images. Qi et al. [2] combined shape description and feature matching, and applied it to trademark retrieval. Anuar et al. [3] improved the performance of trademark retrieval by integrating global descriptors and local descriptors. Considering the rotation invariance of trademark images, Liu et al. [4] proposed a shifting feature matching scheme to improve the effect of trademark retrieval. Toriu et al. [5] proposed a trademark retrieval system based on rotation invariant local features. Feng et al. [6] proposed a set of trademark retrieval process methods by extracting edge features, segmenting images, and using Fisher Vector (FV) to extract enhanced Scale Invariant Feature Transformation (SIFT) features. However, relying on hand-craft features extracted by traditional methods to judge image similarity is too subjective, and similarity has greater deviations due to different levels of influencing factors, such as visual levels or semantic levels associated with images [1]. Therefore, researchers began to conduct trademark retrieval from the perspective of learning deep features.

In recent years, deep features have begun to be applied to trademark retrieval. Tursun [7] applied deep neural network to trademark a feature extraction method for the first time, and experiments showed that its effect was significantly better than traditional methods. In order to make the semantic expression of the image more comprehensive, Wang et al. [8] introduced the Regional Proposal Network (RPN) to extract local features through object proposal regions, and used Faster Region-Convolutional Neural Network (R-CNN) to extract global feature descriptors to obtain a better trademark retrieval result.

At present, most trademark retrieval methods are based on deep learning extract trademark features by a supervised way [7]. Perez et al. proposed a retrieval of trademarks through the combined VGG network [1] with supervised training, Tursun et al. [9] removed the text of trademarks and combined soft and hard attention mechanisms to direct attention to key information. Lan et al. [10] proposed a method to extract uniform Local Binary Pattern (LBP) features from the feature map of each convolutional layer feature, and achieved good results in both METU and NPU trademark datasets. Xia et al. [11] built a deep hash learning framework to learn image binary codes by integrating a spatial transformer network and a recursive convolution network, so as to perform trademark retrieval. However, retrieval work often needs to face a large number of dynamic changes and streaming database images, and the massive amount of data makes the annotation more difficult [12], and it is even infeasible in some fields. Considering the huge amount of trademark data, the unsupervised method is more efficient for retrieval. Wu et al. [12] proposed an extreme unsupervised learning method instance discrimination based on the opinion that the similarity of visual data themselves makes certain classes closer than others.

**Figure 1:**
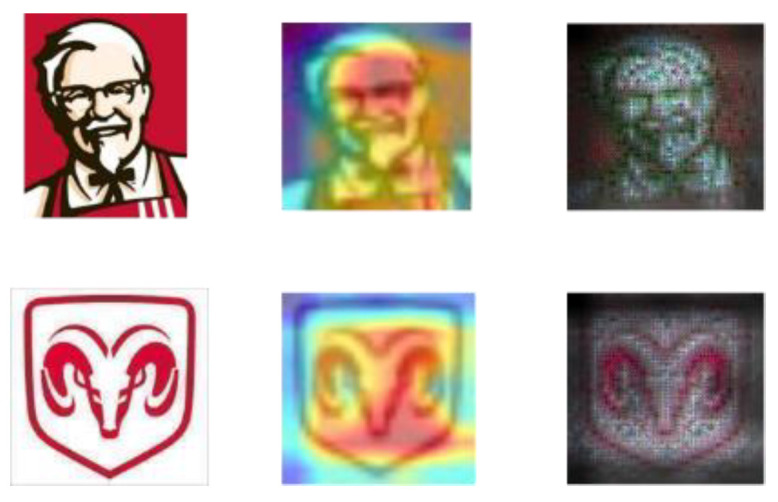
Schematic diagram of attention mechanism in trademark image.

Although unsupervised methods avoid the work of data annotation, they cannot accurately distinguish the key information of image features. Recently, attention mechanisms have received extensive attention in image feature learning. In some respects, trademark images are similar to natural images, and the importance of different areas in the image is usually different. The structure of certain trademarks determines that its own pattern becomes the most important information of the trademark image. For example, some combined trademarks, in which the graphic elements are in the middle, are often more likely to arouse people’s attention. Since introducing the attention mechanism into deep learning has attracted widespread attention and has shown great potential for performance improvement, our work considers whether the attention mechanism can be introduced into trademark retrieval. Compared with the mathematical definition, the attention mechanism is closer to methodology, which adjusts the direction of attention and the weighting model according to specific task goals. Figure 1 shows that after introducing the attention mechanism to the trademark image, the learning of the neural network becomes more targeted. The greater the proportion of red, the clearer the texture of the covered area. That is, the neural network assigns more learning weights to this part of the area. Therefore, our works try to apply the attention mechanism to trademark retrieval to obtain a better representation of trademark features, thereby enhancing the performance of trademark retrieval. Among a large number of attention methods, the most representative one is SENet [13], which learns the channel attention of each convolution block and brings significant performance improvement compared with various deep CNN structures. Wang et al. [14] proposed a lightweight channel attention structure and verified through experiments that the attention model can extract more important features under more efficient and lightweight conditions.

Most of the existing trademark retrieval methods require a large number of labeled samples, which is time-consuming. In order to solve the problem of the high cost of data annotation and the inability to capture key information to improve trademark retrieval performance, this paper proposes an unsupervised trademark retrieval method based on channel attention. In the proposed method, the instance discrimination framework is adopted and a lightweight attention mechanism is introduced to allocate more reasonable learning weight to key features. With the unsupervised way, this proposed method can obtain good feature representation of trademarks and improve the performance of trademark retrieval. Extensive comparative experiments on the METU trademark dataset are conducted. Our experiments show that the trademark retrieval method proposed in the paper is significantly better than traditional retrieval methods, and it is also very competitive compared with other deep learning methods.

## 2. Related Work

### 2.1. Unsupervised Learning

Due to the increase in data volume, unsupervised learning has attracted more attention. As a common unsupervised learning method, self-supervised learning uses the internal structure of data to learn the characteristics of a specific part of an object as accurately as possible when the part of the object information is known. Doersch et al. [15] tried to obtain a better visual representation by combining multiple self-supervised tasks. Although self-supervised learning can capture the relationship between various parts of an example, its related theoretical analysis has not been perfected [16]. As another common unsupervised learning method, generative models are mainly aimed at reconstructing the distribution of data as realistically as possible. In recent years, generative adversarial networks and variational auto-encoder [17,18] have been verified in various fields of research to help improve both generative qualities and feature learning. Donahue et al. [19] proposed to add an encoder that can be any standard convolutional network to extract visual features from Generative Adversarial Nets (GANs). In order to distinguish between real images and generated images, this method also needs to construct generative and discriminative model, which adds a certain amount of work to the training process. Metric learning expresses the relationship between objects by selecting appropriate metric methods, and learns the feature space accordingly. This idea has been widely used in the field of face recognition [20] and person re-identification [21] in a supervised manner. In addition, Dosovitsky et al. [22] trained unlabeled data for unsupervised feature learning. However the method uses parameterized examples, which results in the weights obtained being only valid for the training category, and the generalization is not enough to apply to other categories or instance. Wu et al. [12] proposed an unsupervised feature learning method that can directly distinguish instance categories through a non-parametric classifier.

### 2.2. Attention Mechanism

Deep Convolutional Neural Networks (CNNs) have been widely used in artificial intelligence. Starting from the pioneering AlexNet [23], in order to further improve the performance of deep neural networks, people have begun to conduct related research. In recent years, in order to make computers more adaptable to human communication scenarios, they must be taught to choose forgetting and associated context. So, the attention mechanism is introduced into the corresponding field.

Since the attention mechanism was proposed, an attention module combined with CNNs has become one of the mainstream research methods. With the proposal of the representative channel attention method SENet [13], the network has successfully improved the performance of various CNN architectures by learning the channel attention of each convolution module, which makes the attention mechanism show great potential in network performance. Subsequently, the attention mechanism focuses on enhancing feature aggregation. Convolutional Block Attention Module (CBAM) [24] uses average-pooling and max-pooling to aggregate information. Global second-order pooling convolutional networks (GSoP) [25] made full use of context information in an image by modeling the correlation between the overall tensors. Gather-Excite (GE) [26] introduced a pair of operators, “gather” and “excite “, to capture remote feature interactions and after aggregating feature responses, it can redistribute the combined information to the local area. In addition, the combination of different dimensions of attention became a research hotspot. CBAM and BAM [27] use the channels and spatial dimensions to infer the attention map and learn the characteristics of the data. The difference is that BAM is connected in parallel, while CBAM is connected in series. sequentially inferred the attention map along independent channels and spatial dimensions, and learned the features of the data. After Non-Local (NL) [28] was proposed, self-attention became one of the research hotspots. While maintaining the accuracy of NL, GCNet [29] designs a global context block that can reduce the amount of calculation based on the structure of SENet, which can capture global information more effectively. As a classic self-attention network, DANet [30] combines NL and CBAM to capture the dependencies between different features by adding the attention of channels and spaces. A2-Nets [31] proposes a double attention block for collecting and distributing long-range features, which can model long-range interdependencies with lower computation and memory. Starting from SENet, many of the attention methods derived have achieved excellent performance in various fields, but the application of these modules still has great limitations. Many methods are dedicated to the development of complex attention modules. At the same time, higher accuracy inevitably brings higher model complexity and a heavier computational burden. When dealing with certain huge data tasks, performance improvement may not be enough to cover the negative impact of the complexity increase. Therefore, researchers began to try to build a lightweight neural network architecture without reducing the attention performance.

Most of the existing methods are devoted to developing more complex attention modules to obtain a better performance, which inevitably increases the complexity of the model. The attention module in SENet uses a global average pool independently for each channel firstly, and then uses two Fully Connected (FC) layers with non-linear and a sigmoid function to generate channel weights. The two FC layers are designed to capture non-linear cross-channel interaction, including the use of dimensionality reduction to control the complexity of the model. This idea is widely used in subsequent channel attention methods, such as CBAM and GE, but a large number of experiments have shown that the use of dimensionality reduction methods bring side effects to channel attention prediction [14]. In addition, the introduction of efficient convolution is a conventional method of constructing lightweight CNN architecture, of which group convolution [32] and depth-wise separable [33] convolution are the two most widely used. The paper [14] demonstrated through experiments that this type of convolution involves a small amount of parameters, but the little improvement brought by the application of the attention module.

## 3. The Proposed Method

**Figure 2:**
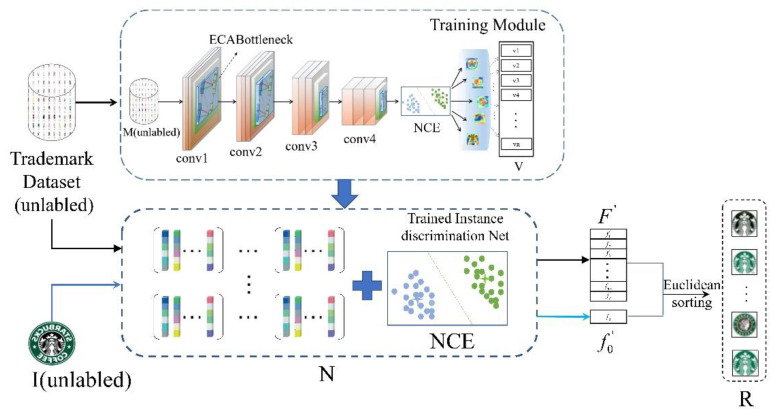
Unsupervised trademark retrieval method with embedded attention module.

Through the above introduction, instance discrimination can perform unsupervised learning on large-scale data under the premise of taking into account the amount of calculation and complexity of calculation. However, relying only on instance discrimination is not enough to make the network achieve an ideal effect on the feature learning of trademark images. Therefore, in order to make neural network focus on key areas to learn trademark channel features, this paper proposes a trademark retrieval method based on the attention mechanism. This method is based on an instance discrimination framework and introduces a lightweight channel attention module that realizes local cross-channel information interaction. While applying unsupervised learning, it uses the attention mechanism to enhance the unsupervised network’s ability to get key channel information and learn a more accurate representation of trademark features. The overall process of this method is shown in Figure 2, which consists of three modules: Channel attention module, unsupervised training module, and retrieval module.

### 3.1. Learning about Important Features of Trademarks

**Figure 3:**
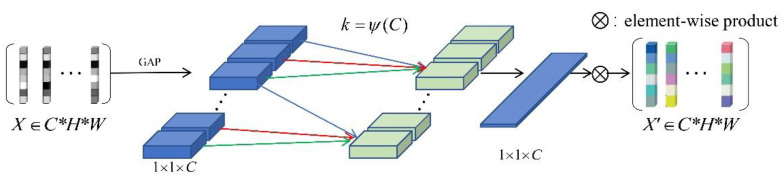
ECA (Efficient Channel Attention) module structure.

In order to overcome the contradiction between performance and complexity, the paper [14] combined the dimensionality reduction and cross-channel interaction, and proposed a channel attention module ECA (Efficient Channel Attention) for deep CNNs. The structure of the module is shown in Figure 3, and only involves a small number of parameters, while bringing significant performance gains. By comparing the traditional channel attention mechanism, it theoretically explains the importance of avoiding dimensionality reduction for learning channel attention, and proper cross-channel interaction can significantly reduce model complexity while maintaining performance. Specifically, the weight of the channel feature $y_{i}$ only considers the association between it and its k neighboring channels:

(1) $$\omega_{i}\;=\;\sigma \left(\sum_{j=1}^{k}\omega_{i}^{j}y_{i}^{j}\right){,\;y}_{i}^{j}\in \Omega_{i}^{k}.$$

In order to further improve performance and make all channels share weight information, the weight calculation method is changed to:

(2) $$\omega_{i}\;=\;\sigma \left(\sum_{j=1}^{k}\omega^{j}y_{i}^{j}\right){,\;y}_{i}^{j}\in \Omega_{i}^{k}.$$

According to the above analysis, the proposed attention module can realize information interaction between channels through 1D convolution with a convolution kernel size of k, and the weight calculation method is finally expressed as:

(3) $$\omega \;=\;\sigma \left({C1D}_{k}\left(y\right)\right)$$

where C1D denotes 1D convolution, and σ() denotes the sigmoid function.

In order to realize the idea that the ECA module can properly capture the local cross-channel information interaction, it is necessary to limit the scope of the interaction information, that is, the value of the convolution kernel size. For the selection of the optimal information interaction range of convolutional blocks with different channel numbers in various neural network structures, a common method is to manually tune cross-validation, but this method consumes a lot of computing resources. Thanks to the successful application of grouped convolution in improving the structure of neural networks, it can be found that under the premise of a fixed number of groups, and the effect of high-dimensional (low-dimensional) channels and long-distance (short-distance) convolution is proportional [32,34,35]. Therefore, the cross-channel interaction range, that is, the value of k should also have a proportional relationship with the channel dimension C. It can be inferred that there is a linear relationship between k and C:

(4) C = ∅(k).

Limited to the limitations of linear functions for certain related features, and the channel dimension is usually an exponential multiple of 2, the relationship between C and k is more reasonably expressed as:

(5) $$C\;=\;\emptyset \left(k\right){\;=\;2}^{\left(\gamma *k-b\right)}.$$

Given the number of channels C, the convolution kernel size k can be calculated by the following formula:

(6) $$k\;=\;\psi \left(C\right)\;=\;{\left|\frac{\log_{2}\left(C\right)}{\gamma }+\frac{b}{\gamma }\right|}_{\mathrm{odd}}$$

where ${\left|x\right|}_{\mathrm{odd}}$ represents the odd number closest to x, and b and γ are set to 1 and 2 respectively.

The attention module introduced in our work generates channel attention through fast 1D convolution. The size of its convolution kernel determines the range of interaction between channels, which can be adaptively determined by the nonlinear mapping of channel dimensions. By avoiding dimensionality reduction and local cross-channel interaction, the ECA module takes into account the learning effect of channel attention while ensuring that the model complexity is not too high.

### 3.2. Instance Discrimination

**Figure 4:**
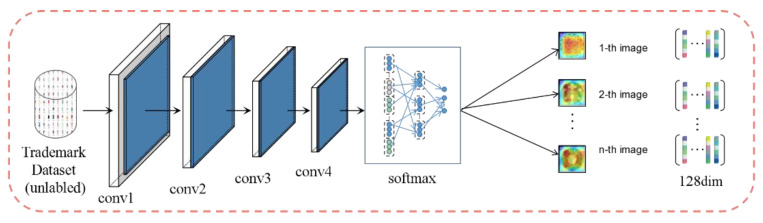
Unsupervised feature leaning approach.

Inspired by the output ranking in supervised learning, Wu et al. [12] points out that the similarity of classes is judged based on the visual data themselves, rather than semantic labels. Based on this, they propose an extreme unsupervised learning method—instance discrimination. This method is “instance-level discrimination”, which treats each image instance as its own category, and then trains a classifier to distinguish different instance categories. The feature learning process of the instance discrimination method is shown in Figure 4. The paper [12] verifies that the application of instance discrimination in classification problems can be significantly improved compared to other methods, and has a positive effect on the learning of image features. Therefore, our work attempts to introduce the unsupervised method of instance discrimination into trademark retrieval.

Instance discrimination method aims to train the neural network to extract image features by distinguishing the difference between instances and noise. The goal is to learn a feature map from unsupervised information:

(7) $${v\;=\;f}_{\theta }\left(x\right)$$

$f_{\theta }\left(x\right)$ is a CNN with θ as the parameter, and v represents the feature of the image x mapping. Suppose there are n-trademark images $\left\{x_{1}{,x}_{2}{,\ldots ,x}_{n}\right\}$ that belong to n classes, among which the corresponding features $\left\{v_{1}{,v}_{2}{,\ldots ,v}_{n}\right\}$, in the traditional parameter softmax, the probability of feature v being judged as the i-th instance:

(8) $$P\left(i|v\right)\;=\;\frac{\exp\left(\omega_{i}^{T}v\right)}{\sum_{j=1}^{n}\exp\left(\omega_{j}^{T}v\right)}.$$

In Formula (8), $\omega_{j}$ is a weight vector for class j and $\omega_{j}^{T}v$ measures how well the feature v matches the category j. For softmax with parameters, because the weights in it hinder the explicit comparison between instances, they cannot be generalized to new categories or new instances. After removing these weight vectors, the learning goal was changed to the feature representation and introduction measurement. This can be applied to any new instance, so a non-parametric softmax method is proposed, replacing $\omega_{j}^{T}v$ with $v_{j}^{T}v$. At the same time, this method eliminates the need for the calculation and storage of the weight vector gradient. Then the probability becomes:

(9) $$P\left(i|v\right)\;=\;\frac{\exp\left(v_{i}^{T}v/\tau \right)}{\sum_{j=1}^{n}\exp\left(v_{j}^{T}v/\tau \right)}$$

and it is equivalent to minimizing its negative log-likelihood:

(10) $$J\left(\theta \right)\;=\;-\sum_{i=1}^{n}\mathrm{logP}\left({i|f}_{\theta }\left(x_{i}\right)\right)\;=\;-\sum_{i=1}^{n}\mathrm{logP}\left(i|v\right).$$

The parameter τ affects the concentration of data distribution [36].

In order to calculate the probability of (9), a feature memory bank V is used to store the features, so as to avoid the problem of excessive calculation caused by calculating the features of all images every time. Assuming that $f_{i}$ is the feature of the image $x_{i},$ input to the network $f_{\theta }$, the stochastic gradient descent algorithm adjust $f_{i}$ and network parameters θ in each learning iteration, and then the features of the corresponding trademark instance stored in V are updated, and $v_{i}$ is updated to $f_{i}$. Since the instance discrimination algorithm regards each picture as a characteristic of an instance, the introduction of the feature storage module cannot completely solve the problem of excessive calculation. When facing a huge amount of image data such as trademarks, the calculation cost of non-parametric softmax is very high. Therefore, Noise Contrast Estimation (NCE) [37] is introduced to convert the multi-classification task into a series of binary classification tasks, that is, to distinguish between data samples and noise samples to solve the problem of excessive calculation caused by calculating the similarity of all instances in the training set, so as to estimate all the classification result of the sample. Specifically, the probability that the feature in the memory bank that corresponds to the i-th category is:

(11) $$P\left(i|v\right)\;=\;\frac{\exp\left(v^{T}f_{i}/\tau \right)}{Z_{i}}$$

(12) $$Z_{i}\;=\overset{n}{\underset{j=1}{\sum }}\exp\left(v_{j}^{T}f_{i}/\tau \right)$$

where $Z_{i}$ is the regularization constant in the above formula. On the premise that the noise sample is m times the data sample and the noise distribution $P_{n}$ is set to a uniform distribution, the posterior probability of the feature v corresponding to the sample i is:

(13) $$h\left(i,\;v\right):\;=\;P\left(D\;=\;1|i,\;v\right)\;=\;\frac{P\left(i|v\right)}{P\left(i|v\right){+\mathrm{mP}}_{n}\left(i\right)}$$

(14) $$P_{n}\;=\;1/n.$$

The goal of training is to make the negative log-posterior distribution of trademark instances and noise samples in the trademark dataset as small as possible:

(15) $$J_{\mathrm{NCE}}\left(\theta \right){\;=\;-E}_{P_{d}}\left[\mathrm{logh}\left(i,\;v\right)\right]{-\mathrm{mE}}_{P_{n}}\left[\log\left(1-h\left({i,\;v}^{\prime }\right)\right)\right].$$

Both v and v’ are sampled from the non-parameter feature memory bank V, which stored the features obtained by unsupervised training.${\;P}_{d}$ denotes the actual data distribution. For $P_{d}$, v is the feature of the trademark image $x_{i}$ as an instance. In contrast, v’ is the feature of the noise sample different from the instance image obtained by sampling according to the distribution of $P_{n}$.

The overall process of instance discrimination in trademark retrieval:

1. Select training samples from the trademark database and preprocess them to obtain $X\;=\;\left\{X_{i}\right\},i\;=\;1,2\cdots n$, form training batches;
2. Input the training set into the unsupervised network, extract the features to get the initial feature set $V\;=\;\left\{v_{i}\right\}$, and store it as the corresponding feature of the current batch;
3. Sample negative samples from the stored feature set s;
4. Calculate the loss value of the instance sample and the noise sample collected from the memory bank;
5. Use back propagation to continuously optimize the target value and update the parameters until the end of the training.

In general, instance discrimination extends the view that visual similarity is learned from the visual data themselves to unsupervised learning. It applies class-level discrimination information to the extreme, and treats each image as a separate instance. The method makes the features of a single instance discriminative, so as to learn better feature representations and capture the similarities between instances rather than classes. In addition, the introduction of the NCE method has transformed multi-classification tasks into two-class classification tasks, greatly reducing the computational complexity of the instance discrimination under big data conditions, and the computational complexity has been reduced from O(n) per sample to O(1), which makes it possible to apply the method of instance discrimination in large-scale trademark data.

### 3.3. Similarity Measure

In order to judge the similarity between trademark images, we first extract the feature vector of the trademark to be retrieved. Then extract the feature vector of the trademark database or other trademark images that need to be compared, and calculate the similarity score by dot product to determine the similarity:

(16) $$\mathrm{sim}\left(a,\;b\right)\;=\;\mathrm{dot}\left(a,\;b\right){\;=\;a}^{T}\cdot b$$

where a and b represent the feature vectors corresponding to the two compared trademarks, respectively.

### 3.4. The Process of Our Proposed Method

Our work is based on the instance discrimination framework and introduces the channel attention module. The purpose is to help the neural network more accurately capture the channel feature information of the input image, allocate more computing resources to the detailed information of the target that needs attention, and suppress other useless or unimportant information. It can obtain a more reasonable weight distribution. As shown in Figure 3, after global average pooling in the channel without reducing the dimensionality, the ECA module captures local cross-channel interaction information by considering each channel and its k neighbors. The size of the convolution kernel k is the cross-channel interaction. The coverage rate is determined adaptively according to channel dimension C. The unsupervised training module uses unsupervised learning to train the trademark feature extractor through the weights assigned by the attention module. As shown in Figure 2, the training network uses ResNet50 [38] as the backbone model, and at the same time replaces the non-parametric classifier with the NCE module, and embeds the ECA module in it. Conv1~conv4 represent the convolutional layer of the residual network. The number of channel attention blocks corresponding to different network layers are embedded between the two layers. In the training phase, the trademark image samples are input into the network according to the set training batch. The ResNet50 network embedded with the attention module is trained. The feature V of the batch is extracted and stored, and then the backpropagation algorithm is used to calculate the loss and optimize it. V is continuously updated by updating Formula (13), thereby minimizing the objective function of Formula (15). The retrieval module is responsible for calculating the feature similarity and outputting the result. Firstly, the trademark dataset is input to the trained feature extraction network to obtain the trademark feature library, and then the trademark to be retrieved is input to the network to extract the corresponding features of the trademark. Finally, the experiment evaluates their similarity by calculating the Euclidean distance, and outputs the retrieval results according to the distance from small to large. The proposed trademark retrieval process is shown in Algorithm 1.
**Algorithm1:** Unsupervised trademark retrieval method based on attention mechanism**Input:** Retrieved image I, Trademark database M. **Output:** Image sequence R which is similar to I. **Step1:** for i←1 to maximum_epochs do
      1. 
$\mathrm{Select}\;\mathrm{training}\;\mathrm{samples}\;\mathrm{from}\;M\;\mathrm{to}\;\mathrm{obtain}\;\mathrm{the}\;\mathrm{training}\;\mathrm{batches}\;\mathrm{as}\;X=\left\{X_{i}\right\},i=1,2\cdots n$.
      2. $\mathrm{Feature}\;\mathrm{extraction}\;\mathrm{obtains}\;v_{i}$$\;\mathrm{to}\;\mathrm{form}\;\mathrm{feature}\;\mathrm{set}\;V=\left\{v_{i}\right\}$, put V into the instance discrimination module.      3. 
$\mathrm{Calculate}\;\mathrm{the}\;\mathrm{loss}\;\mathrm{from}\;v_{i}$ and optimize loss, update V iteratively.
      4. Backpropagate the loss and update the parameters.
      5. Repeat the above steps until the algorithm converges to get the feature extraction network N.
   end for **step2:**      1. $\mathrm{Put}\;M\;\mathrm{into}\;\mathrm{the}\;N,\;\mathrm{form}\;a\;\mathrm{database}\;\mathrm{of}\;\mathrm{trademark}\;\mathrm{image}\;\mathrm{feature}\;\mathrm{as}\;F^{\prime }=\left\{f_{1}^{\prime }{,f}_{2}^{\prime }\cdots f_{N}^{\prime }\right\}{,F}^{\prime }\in R^{N\times 128}$$,\;\mathrm{store}\;F^{\prime }$ in the retrieval module.
      2. $\mathrm{Put}\;I\;\mathrm{into}\;\mathrm{the}\;N,\;\mathrm{get}\;a\;\mathrm{image}\;\mathrm{feature}\;\mathrm{as}\;f_{0}^{\prime }\in R^{1\times 128}$$,\;\mathrm{store}\;f_{0}^{\prime }$ in the retrieval module.
      3. 
$\mathrm{Measure}\;\mathrm{similarity}\;\mathrm{between}\;F^{\prime }$$\;\mathrm{and}\;f_{0}^{\prime }$, output similar image sequence R.

## 4. Experiment

### 4.1. METU Dataset

**Figure 5:**
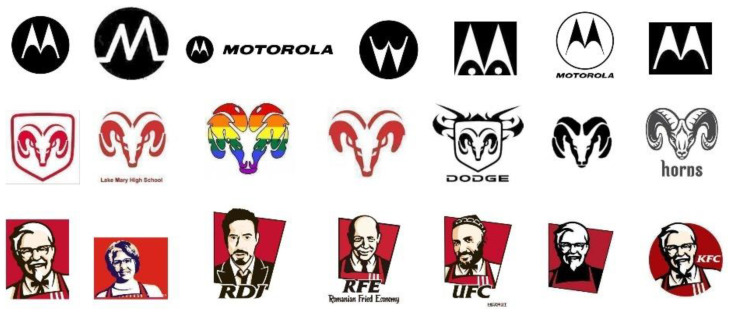
Some images from three groups in the query set.

The METU dataset [7] is currently the largest publicly available trademark dataset that does not require any preprocessing. It makes the expansion of trademark retrieval no longer limited by the number of images and query types. The dataset contains a total of 923,343 trademark related images, and contains three types of trademark images of figures only, text only, and figures with text. The dataset is divided into two parts: The training set and the query set, which are used to learning the model and evaluate the method. The training set contains 922,926 unlabeled trademark images, and the query set consists of 417 trademark images that divided into 35 groups, with 10–15 similar trademarks in one group. The images contained in the query set are extremely challenging for existing computer vision and image retrieval methods, as shown in Figure 5. In the experiment, all trademark images are set to a uniform size of 32 × 32.

### 4.2. Evaluation Method and Metrics

In information retrieval, precision and recall are often used to measure performance. Taking into account the large amount of trademark data, this paper uses NAR (Normalized Average Rank) [7] to evaluate the effect of trademark retrieval. As a normalized index, it can make the calculation result avoid the impact of the size of the database and the data to be retrieved as much as possible. Similar to mAP (Mean Average Precision), it comprehensively considers the reconciliation of precision and recall to a certain extent, and is a comprehensive indicator. The calculation formula of NAR is:

(17) $$\mathrm{NAR}\;=\;\frac{1}{{N*N}_{\mathrm{rel}}}\left(\overset{N_{\mathrm{rel}}}{\underset{i=1}{\sum }}R_{i}-\frac{N_{\mathrm{rel}}\left(N_{\mathrm{rel}}+1\right)}{2}\right)$$

where N is the size of the dataset, $N_{\mathrm{rel}}$ is the number related to the image to be retrieved, and $R_{i}$ represents the ranking of the image related to the image to be retrieved in the result. Based on the use of NAR, in order to test the stability of the algorithm, the MSE (Mean Squared Error) is introduced as another evaluation index. The values of NAR and MSE are inversely proportional to the retrieval performance and stability of the algorithm, the smaller the value of the two, the better the retrieval performance and stability of the algorithm.

### 4.3. Experimental Settings

#### 4.3.1. Training Parameters

In this paper, the work of unsupervised learning of trademark features is completed by using instance discrimination framework. Considering the expected performance and cost consumption, our experiment adopts the ResNet50 network as the backbone network for the experiment, and the specific parameters of the training and testing phases are consistent with the paper [1,12,14]. The learning rate is set to 0.03. The *k* value is set to be determined by the adaptive method. The temperature parameter in Formula (9) is set to 0.07, and the value of m in Formula (13), that is, the sampling multiple of noise sample contrast and data sample, is set to 4096. In training, the dimension of the feature is set to 128, the batch of the training set is 256, and the batch of the query set is 100. In specific experiments, we found that when the number of training is 120, the training loss has reached a stable threshold. Although continues learning can reduce the training loss, it also causes overfitting. Therefore, we set the number of training to 120 times, and the algorithm at this stage has tended to converge to meet the needs of the algorithm.

#### 4.3.2. Effect of *k* on ECA Module

**Figure 6:**
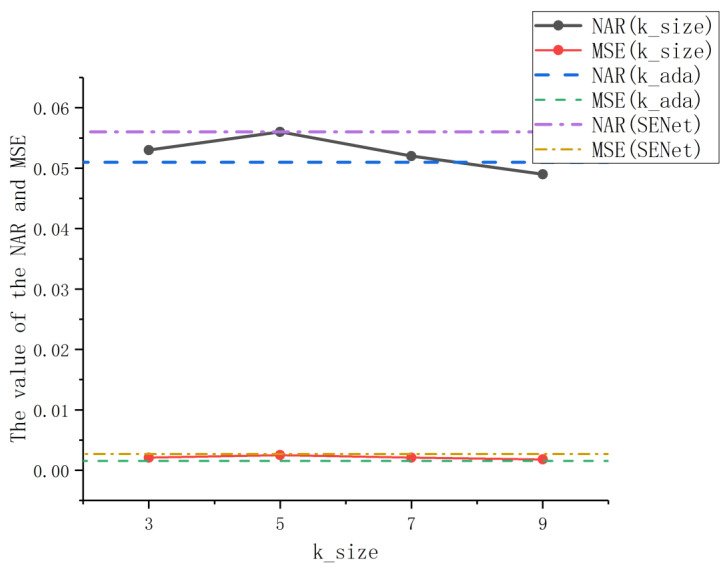
The performance of trademark retrieval results under different *k* values. NAR: Normalized Average Rank; MSE: Mean Squared Error.

As shown in Formula (3), the ECA module involves a parameter *k*, that is, the kernel size of 1D convolution, which affects the coverage of cross-channel interaction. In this part, we evaluate the influence of the *k* value on the ECA module. The experiment uses ResNet50 as the backbone network, corresponding to the adaptive selection formula of the *k* value, and sets *k* to an odd value between 3 and 9 for the experiment. The experimental results are shown in Figure 6.

In the line chart, the solid lines represent the results obtained by manually fixing the *k* value, the dotted lines represent the results obtained by adjusting the *k* value through an adaptive method, and the dash-dotted lines represent the results obtained by the SENet model experiment. It can be seen from the solid line that the value of *k* has a significant impact on the performance of the ECA module. When *k* = 9, because the channel exchanges more information, the algorithm with ResNet50 as the backbone network achieves the best retrieval effect, and the corresponding NAR and MSE values are the lowest. Combined with the result comparison of the dashed line, the adaptive kernel size avoids the manual adjustment of parameter *k* through cross-validation, while obtaining a NAR value close to the optimal effect, and is better than the result of a fixed *k* value in the MSE value. At the same time, by comparing with the dot-dash line representing the retrieval results of the SENet method, it can be observed that the solid and dotted lines with different *k* values are not higher than the dot-dash line, indicating that the performance of the ECANet method in trademark retrieval is better than the SENet method. It is proved that avoiding dimensionality reduction and introducing local cross-channel interaction on the basis of the SENet model have a positive effect on the learning of key trademark features. Therefore, this experiment verifies that the adaptive kernel size selection used in this paper is effective in trademark retrieval.

### 4.4. Experimental Results and Analysis

In order to verify the effectiveness of the proposed method, the paper compared with the effect of traditional methods and deep learning methods in trademark retrieval.

#### 4.4.1. Compared with Traditional Feature Extraction Methods

Refer to papers [1,7], this paper selects the traditional feature extraction methods commonly used in trademark retrieval, including Color Histogram (CH) [39], Local Binary Pattern (LBP) [40], Generalized Search Tree (GIST) [41], Shape Context (SC) [42], Scale Invariant Feature Transformation (SIFT) [43], Speeded Up Robust Features (SURF) [44], Histogram of Oriented Gradient (HOG) [45], Orientation-Restricted SIFT (OR-SIFT) [46], and so on. The above results are from the paper [1]. The relevant data and evaluation indicators used in our experiment are consistent with the paper. The experimental results are shown in Table 1. It can be observed that the method proposed in this paper is compared with traditional feature extraction methods and have been significantly improved. As traditional methods focus more on shallow features, it is impossible to assign the weights of feature learning comprehensively or with emphasis on trademark images with rich information elements. Different from this, this paper introduces the channel attention mechanism based on the learning of in-depth features, so it can learn the deep channel features of trademark images more targeted, so as to extract trademark features more accurately, and improve the effect of trademark retrieval.

#### 4.4.2. Compared with Deep Learning Methods

The experiment refers to the supervised feature extraction methods in the paper [1], including mainstream deep neural networks such as AlexNet [23], GoogLeNet [47], VggNet [48], and ResNet. In addition, we also compare with the attention models commonly used in recent years, including the classic model SENet in the channel attention field, the model CBAM which combines the channel and the space field, and the representative group convolutional structure neural network SKNet [49], the residual network ResNeXt [34] combined with SENet through which grouped convolution is introduced, and so on. In the table, AlexNet (FC7) indicates that the FC7 layer of AlexNet is used to extract features, and other networks are the same. It can be seen from the experimental results in Table 2 that compared with the traditional methods in Table 1, after the introduction of deep learning, the effect of trademark retrieval has been significantly improved. Since the VGG network has learned more general representations than other networks [7], the effect is better. In addition, VGG19v and VGG19c can complement each other in the classification task [1], which makes the combination of the two further improve the result of trademark retrieval. CNN does not pay much attention to key information in the trademark image, and the deep learning method that introduces the attention mechanism solves the problem that the corresponding network cannot flexibly and specifically capture the key features of the trademark image in the learning stage. The method proposed in the paper combines a lightweight attention network that can realize local cross-channel information interaction. The network takes into account the integrity of feature information and local channel interaction, so that the network focus on the features of trademark images is more flexible, thereby improving the accuracy of feature capture. At the same time, combining with the unsupervised learning algorithm instance discrimination, can avoid labeling a large amount of data, saving a lot of manpower and time, and the retrieval effect obtained on this basis is better than most supervised methods. Considering cost and performance, the proposed method has obvious advantages.

In order to compare the retrieval effect of the method in this paper with the residual network without introducing attention more intuitively, four trademark images are selected randomly as the query to retrieve. Then the features of the query and the top five retrieved trademarks are extracted and the similarities are computed. The closer the score is to 1, the more similar the results are. The average similarity scores of the top 5 and their averages are recorded as shown in Table 3. In Table 3, the US represents our method, RES denotes the ResNet50 network, and the suffix of the method stands for the number of ranking. It can be seen that the similarity score obtained by our proposed method is closer to 1 than ResNet, which verifies that our method has an advantage in judging trademark similarity.

#### 4.4.3. Visualization of the Results

**Figure 7:**
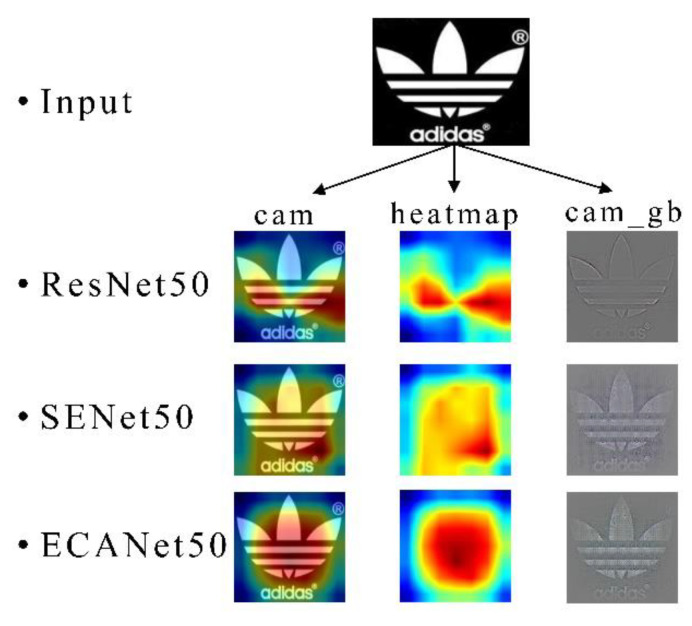
Visualization effect of Convolutional Neural Network (CNN) on trademark image.

In order to more clearly illustrate the effectiveness of the method proposed in the paper in the application of trademarks, this section presents the effect of retrieval in a visual way. The three rows shown in Figure 7 are the effects of the ResNet50, SENet, and ECANet networks acting on the trademark to obtain information. The CAM (Class Activation Map) [50] in the first column can indicates the sensitive relationship between the regional pixels in the picture and the output probability by temperature. The sensitivity is directly proportional to the temperature, that is, the greater the proportion of red, the more attention the network pays to the area. The second column of heat maps concealing trademark images can more intuitively observe the sensitive areas of the network. In the third column, we visualize the capture of features from the perspective of image texture. From the comparison of ResNet50, SENet, and ECANet, it can be found that the introduction of attention makes the network capture more key information of trademark images for feature learning. In a comparison between ECANet and SENet, it can be observed that because ECANet avoids dimensionality reduction, it retains more important information, benefiting from the local cross-channel interaction, ECANet’s visualization effect shows the coverage of more key information in the trademark image, so a better feature extraction model can be obtained through training.

**Figure 8:**
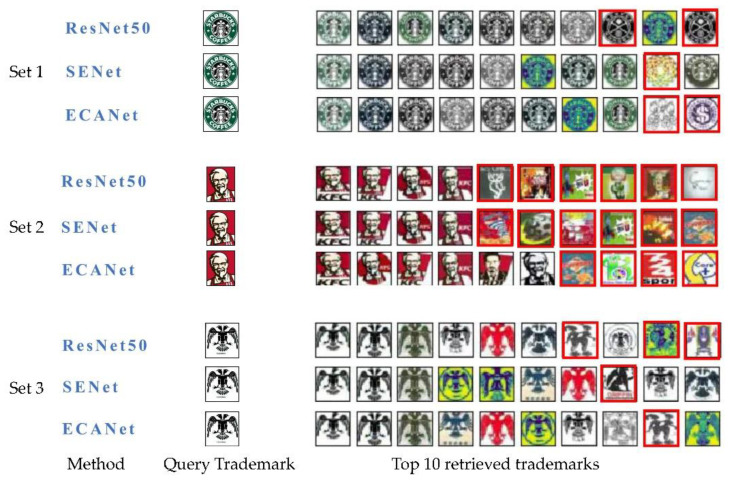
Comparison of trademarks retrieved by ResNet50, SENet, and ECANet.

In addition, the retrieval results with three query trademarks selected randomly on the METU dataset are shown in Figure 8. As shown in Figure 8, the first column images are the query images, and the last 10 columns are the corresponding retrieval trademarks sorted from high to low in terms of similarity. Each query trademark is fed to three representative methods respectively, so there are three groups. Each row consists of the query trademark and retrieved trademarks by one method. In the comparison of the ranking results, there is almost no difference in the high-ranking results obtained by the three methods. When the similarity decreases, the difference arises. The incorrect results are highlighted by the red rectangular boxes. In the first group, the 8th and 10th query results of ResNet50 are wrong, and the 9th query result of SENet is wrong. The 9th and 10th retrieved trademark of ECANet50 are wrong. It is noted that only ECANet50 can find similar trademarks in the last five retrieved results in the second group. Similarly, in the third group, there are 3, 1, and 1 mistake trademarks retrieved by Resnet50, SENet, and ECANet, respectively, and ECANet50 obtained the more backward position of a mistaken trademark than SENet, which means better retrieval performance. In Figure 8, although there are some mistakes in the retrieval results of these methods, ECANet can generally retrieve more correct trademark images than other methods. It is intuitively verified that the introduction of the ECA module is effective in improving trademark retrieval.

## 5. Conclusions

To solve the problem of the high cost of data annotation and insufficient attention to important channel features, the paper introduced a lightweight attention network that realized local cross-channel interaction into an instance discrimination framework for trademark retrieval. This method assigns more reasonable weights to key features from the perspective of focusing and associating the important channel information of the image to obtain more accurate feature representation. Experiments on the METU dataset showed that the performance of the method proposed was better than traditional trademark retrieval methods and most existing supervised methods, verifying the effectiveness and feasibility of our proposed method in trademark retrieval. In future, we will try to verify the feasibility of applying the self-attention mechanism to trademark retrieval and further study the combination of unsupervised learning and trademark retrieval.

## Figures and Tables

**Table 1:** Comparison results with traditional trademark retrieval methods.

Method	NAR ± MSE
CH ^1^	0.400 ± 0.175
LBP ^2^	0.276 ± 0.142
GIST ^3^	0.254 ± 0.173
SC ^4^	0.220 ± 0.186
HOG ^5^	0.262 ± 0.129
SIFT ^6^	0.179 ± 0.145
OR-SIFT ^7^	0.190 ± 0.151
SURF ^8^	0.207 ± 0.151
Our Method	0.051 ± 0.002

^1^ Color Histogram. ^2^ Local Binary Pattern. ^3^ Generalized Search Tree. ^4^ Shape Context. ^5^ Histogram of Oriented Gradient. ^6^ Scale Invariant Feature Transformation. ^7^ Orientation-Restricted SIFT. ^8^ Speeded Up Robust Features.

**Table 2:** Comparison with deep learning trademark retrieval methods.

Method	NAR ± MSE
ResNet50 (FC1000)	0.110 ± 0.133
ResNet50 (Pool5)	0.095 ± 0.138
VGGNet16 (FC7)	0.086 ± 0.107
AlexNet (FC7)	0.112 ± 0.171
GoogleNet (77S1)	0.118 ± 0.138
VGG19v	0.066 ± 0.130
VGG19c	0.063 ± 0.128
VGG19v + VGG19c	0.047 ± 0.095
SENet	0.056 ± 0.003
SENet (ResNeXt)	0.055 ± 0.008
SKNet	0.068 ± 0.002
CBAM	0.056 ± 0.003
ResNet50 (dim = 128)	0.063 ± 0.002
Our Method	0.051 ± 0.002

**Table 3:** Similarity score of trademarks in the same class.

Score Index	Pic1	Pic2	Pic3	Pic4
US_1	0.837	0.802	0.881	0.894
US_2	0.821	0.744	0.824	0.731
US_3	0.692	0.673	0.803	0.625
US_4	0.667	0.661	0.752	0.612
US_5	0.655	0.606	0.670	0.580
RES_1	0.860	0.712	0.778	0.807
RES_2	0.734	0.654	0.773	0.579
RES_3	0.667	0.617	0.767	0.497
RES_4	0.605	0.560	0.694	0.426
RES_5	0.570	0.553	0.545	0.415
US_AVG	0.734	0.697	0.786	0.688
RES_AVG	0.687	0.619	0.711	0.545

## Data Availability

Not applicable.
